# A Case Report of Synchronous Multicentric Breast Carcinoma With Biologically Discordant Phenotypes: Luminal A and Triple-Negative Subtypes

**DOI:** 10.7759/cureus.89666

**Published:** 2025-08-09

**Authors:** Maksym K Gmur, Michal Gajewski, Weronika Goliat, Konrad Haraziński, Przemyslaw R Dalski-Szelag, Izabela Jastrzebska, Barbara Sławińska, Nicole Maryniak, Oliwia Sysło, Zuzanna Błecha, Nikola Rubik

**Affiliations:** 1 Medicine, Provincial Hospital in Poznań, Poznań, POL; 2 Internal Medicine, Międzyleski Specialized Hospital in Warsaw, Warsaw, POL; 3 Medicine, Medical University of Silesia in Katowice, Katowice, POL; 4 Medicine, Zagłębiowski Oncology Center in Dąbrowa Górnicza, Dąbrowa Górnicza, POL; 5 Faculty of Medicine, Academy of Silesia, Katowice, POL; 6 Faculty of Medicine, Medical University of Silesia in Katowice, Katowice, POL; 7 Medicine, Academy of Silesia, Katowice, POL

**Keywords:** intertumoral heterogeneity, luminal a subtype, synchronous multicentric breast cancer, tnbc, vacuum-assisted breast biopsy

## Abstract

We present the case of a 45-year-old Caucasian woman diagnosed with synchronous bicentric breast cancer of differing molecular phenotypes in the same breast. The first tumor, an invasive ductal carcinoma (G1), was estrogen and progesterone receptor-positive and HER2-negative, with a low proliferative index (Ki67 10%). A second lesion, located in a different quadrant and appearing within weeks after biopsy, exhibited a triple-negative phenotype and a higher proliferative index (Ki67 30%). Both tumors were classified as stage IA. Due to the multicentric nature of disease, the patient underwent mastectomy with sentinel node biopsy and immediate reconstruction. Genetic testing was negative for BRCA1/2, CHEK2, and PALB2 mutations, though familial cancer history suggested a possible hereditary syndrome (FCC suspicion). This case underscores the complexity of managing multicentric breast cancer with discordant receptor status and raises questions about the role of biopsy sites in tumor development.

## Introduction

Breast cancer is a biologically heterogeneous disease, comprising various histological subtypes and molecular profiles that impact both prognosis and therapeutic strategy. In daily clinical practice, the most commonly encountered form is unifocal carcinoma; however, multicentric (MC) and multifocal (MF) presentations defined, respectively, as the presence of multiple tumor foci in different or same quadrants of the breast are not uncommon, with an incidence ranging from 6% to 75% depending on definitions and imaging techniques used [1,2].

The pathological and molecular evaluation of each lesion in MF or MC breast cancer remains a subject of ongoing debate. Current clinical guidelines often recommend assessing only the largest or most representative tumor for receptor status and molecular subtype [3]. However, increasing evidence points to significant intertumoral heterogeneity in such cases, including discordant expression of hormone receptors, HER2, and proliferation indices between synchronous tumor foci, which may lead to treatment undertreatment or overtreatment if not fully characterized [4,5].

In particular, cases presenting with synchronous tumors that differ substantially in biological behavior, such as the coexistence of a triple-negative breast cancer (TNBC) and a luminal A subtype, pose diagnostic and therapeutic challenges. TNBC is associated with aggressive clinical behavior and poor prognosis, while luminal A tumors tend to be more indolent and responsive to hormone therapy [6,7]. A few studies have documented such discordant synchronous tumors within a single breast, but these remain rare and underreported [5,8,9].

Emerging research emphasizes the importance of evaluating all distinct foci in MC/MF tumors to guide staging and optimize adjuvant therapy decisions. For instance, in a recent study, discordant receptor status was found in up to 25% of bifocal breast cancers, with approximately 8% showing clinically actionable differences requiring modification of systemic treatment plans [4]. Another prospective study suggested that in 13.5% of cases, secondary tumors - not the index tumor - were more biologically aggressive, necessitating a shift in staging and therapeutic prioritization [5].

In this report, we describe a rare case of synchronous MC breast carcinoma in a single breast, consisting of two distinct lesions: one luminal A subtype and the other triple-negative. We discuss its diagnostic journey, implications for management, and contextualize it within current literature addressing biological heterogeneity in breast cancer.

This case is clinically significant as it highlights the diagnostic and therapeutic complexities associated with biologically discordant MC breast carcinomas, which remain underreported despite their potential impact on treatment decisions and prognosis.

## Case presentation

A 45-year-old Caucasian woman presented with a history of hypothyroidism, diagnosed at age 25, and prior hysterectomy performed at age 37 for uterine fibroids and cervical intraepithelial neoplasia (CIN 3). She reported no smoking or alcohol consumption and maintained a normal body mass index. Medical history notable for childbirth at age 22 and breastfeeding lasting two years. The family history was notable for colorectal cancer in her father and breast cancer in two maternal cousins (histological details unavailable). Since 2007, the patient had attended regular follow-up visits every 6 to 12 months at the same outpatient clinic, which included clinical evaluation, physical examination, and imaging (ultrasound and mammography) due to persistent benign-appearing lesions (BI-RADS 2-3).

In February 2021, image-guided vacuum-assisted breast biopsy (VABB) was performed on a suspicious lesion located in the lower inner quadrant (7 o’clock position) of the left breast. Histopathological analysis revealed invasive ductal carcinoma, not otherwise specified (NOS), grade 1, with strong estrogen and progesterone receptor expression (ER+/PR+ >90%), HER2-negative status, and a low Ki-67 proliferation index (10%). Approximately five weeks later, the patient detected a new, firm, non-mobile, painless mass in the upper inner quadrant of the same breast. Subsequent breast ultrasound in March 2021 revealed a second suspicious lesion (11 o'clock position), along with features suggestive of neoplastic infiltration in the area of the prior biopsy cavity.

Given the MC nature of the findings, the previously planned breast-conserving surgery (BCT) was abandoned, and the patient underwent left-sided mastectomy with sentinel lymph node biopsy (SLNB) and expander implantation on April 13, 2021. Histopathological examination confirmed the presence of two biologically distinct carcinomas: 1) invasive ductal carcinoma, grade 1, ER+/PR+ (>90%), HER2-negative, measuring 0.9 cm, Ki-67 index 10%; and 2) invasive ductal carcinoma, grade 3, triple-negative phenotype (ER-/PR-/HER2-), measuring 1.7 cm, Ki-67 index 30%.

All three sentinel lymph nodes were free of metastasis on intraoperative and postoperative evaluation. Both tumors were staged as pT1N0, corresponding to stage IA under the AJCC TNM system. Complete resection R0 was achieved.

Given the presence of a TNBC subtype, the patient received adjuvant chemotherapy consisting of four cycles of doxorubicin and cyclophosphamide (AC), followed by four cycles of docetaxel. Prophylactic granulocyte colony-stimulating factor was administered from the second cycle onward. In April 2022, she underwent expander exchange with final prosthesis implantation. The patient continues hormonal therapy with tamoxifen (20 mg daily) for the ER-positive component of the disease, with the dose maintained consistently throughout the treatment period.

Given her clinical presentation and family history, the patient was referred for genetic counseling. Initial genetic testing and extended multigene panel testing via next-generation sequencing (NGS) revealed no disease-associated mutations in *BRCA1*, *BRCA2*, *PALB2*, or *CHEK2 *(Table 1). Although no abnormalities were detected in the analyzed genes, based on the available clinical data, medical history, and pedigree analysis, features consistent with familial colorectal cancer syndrome were identified, with a high probability of diagnosis and malignant tumor aggregation in the family (cancer family aggregation [CFA]). Therefore, ongoing surveillance and preventive strategies were recommended for the patient’s relatives, with the understanding that currently untested genetic loci may still underlie her cancer predisposition.

**Table 1:** Genetic testing results. ASA-PCR, allele-specific amplification polymerase chain reaction; MLPA, multiplex ligation-dependent probe amplification; multiplex-PCR, multiple primer polymerase chain reaction; NGS, next-generation sequencing; qPCR-HRM, quantitative polymerase chain reaction with high-resolution melting; RFLP-PCR, restriction fragment length polymorphism polymerase chain reaction

Gene	Mutation Analyzed (Type)	Testing Method	Result	Reference Sequence No.
BRCA1	C61G, c.181T>G	ASA-PCR, RFLP-PCR	No alteration detected	NM_007294.3
	4153delA, c.4035delA	qPCR-HRM	No alteration detected	NM_007294.3
	5382insC, c.5266dupC	ASA-PCR	No alteration detected	NM_007294.3
	185delAG, c.68_69delAG	qPCR-HRM	No alteration detected	NM_007294.3
	3819del5, c.3700_3704delGTAAA	qPCR-HRM	No alteration detected	NM_007294.3
	All coding exons + flanking introns (up to 30 bp)	NGS+MLPA	No pathogenic variants detected	NM_007294.4
BRCA2	All coding exons + flanking introns (up to 30 bp)	NGS+MLPA	No pathogenic variants detected	NM_000059.4
CHEK2	c.1100delC	ASA-PCR	No alteration detected	NM_007194.3
	IVS+1G>A	qPCR-HRM	No alteration detected	NM_007194.3
	del5395bp	multiplex-PCR	No alteration detected	NM_007194.3
	c.470T>C, I157T	qPCR-HRM	No alteration detected	NM_007194.3
	All coding exons + flanking introns (up to 30 bp)	NGS	No pathogenic variants detected	NM_007194.4
PALB2	c.172_175delTTGT	qPCR-HRM	No alteration detected	NM_024675.3
	c.509_510delGA	qPCR-HRM	No alteration detected	NM_024675.3
	All coding exons + flanking introns (up to 30 bp)	NGS	No pathogenic variants detected	NM_024675.3

**Figure 1:**
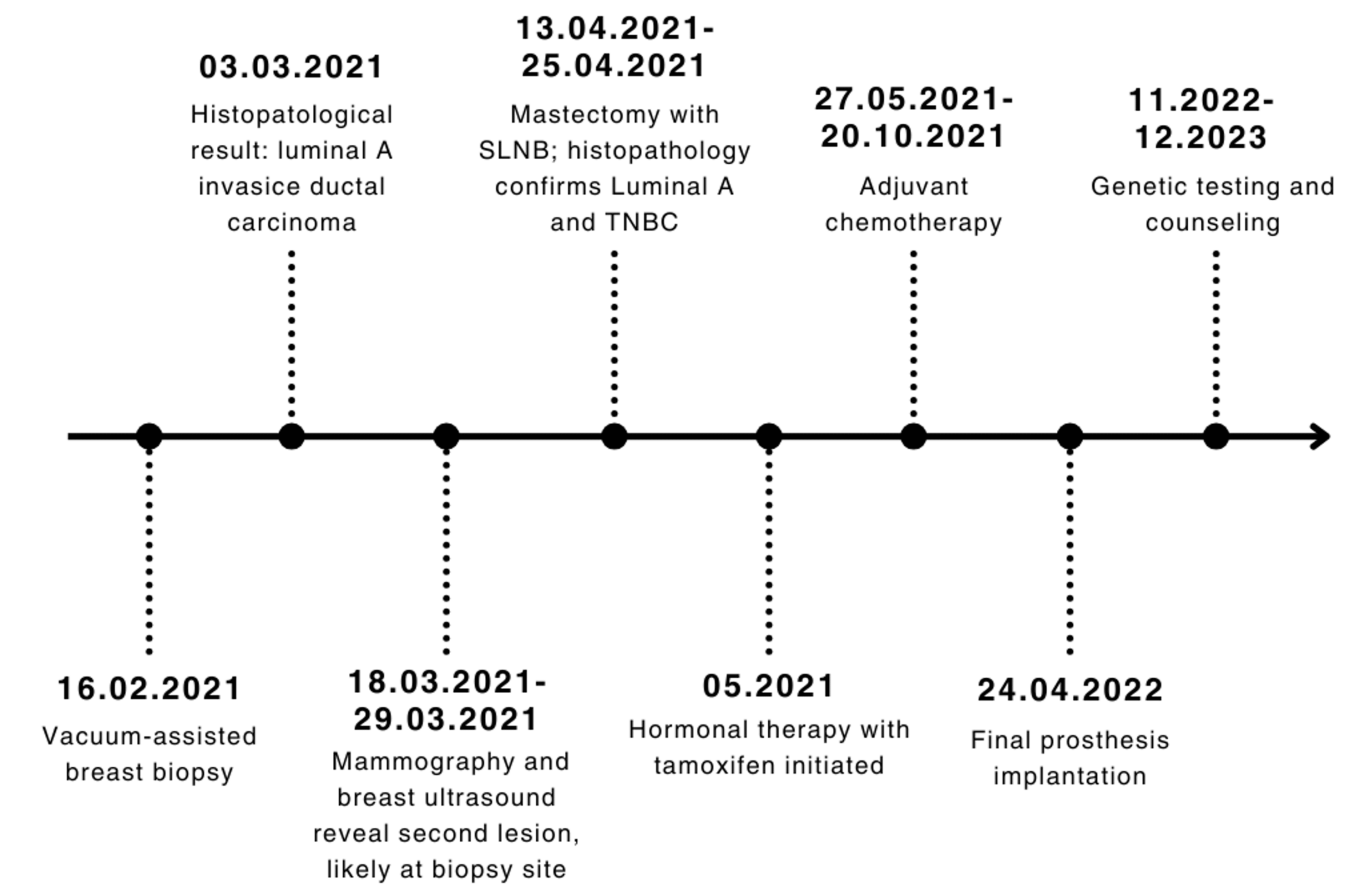
Timeline of clinical course. SLNB, sentinel lymph node biopsy; TNBC, triple-negative breast cancer

At present (July 2025), the patient remains in clinical remission with no evidence of recurrence on imaging or physical examination. She adheres to regular oncologic follow-up and continues hormonal therapy without significant complications. The timeline of the patient’s clinical course is presented in Figure 1.

## Discussion

MF and MC breast cancers are defined by the presence of two or more distinct malignant foci within the same breast. MF tumors are located within the same quadrant, whereas MC tumors are found in different quadrants or are separated by at least 4-5 cm of normal breast tissue [10]. The case presented here, involving two histologically distinct tumors in separate quadrants of the left breast, is a classic example of synchronous multicentric breast cancer (SMBC), a rare yet clinically significant form of breast cancer.

The incidence of MF/MC breast cancer varies between 10% and 24%, depending on imaging modality and diagnostic criteria used [2,7]. Magnetic resonance imaging (MRI) tends to identify additional lesions more sensitively, potentially revealing synchronous tumors that would otherwise remain occult. MC breast cancer is more frequently diagnosed in younger patients and in association with more aggressive subtypes such as TNBC [7]. In the cohort studied, 21.6% of patients with SMBC exhibited molecular subtype discordance between tumors, underlining the biological complexity of this disease [5].

In our patient, the first tumor was a low-grade, hormone receptor-positive, HER2-negative lesion (luminal A phenotype), while the second was a high-grade TNBC with a higher Ki-67 index. Such phenotypic discordance may suggest either clonal divergence or independent tumorigenesis. Djordjevic-Jovanovic et al. demonstrated that discordance in receptor expression between multiple synchronous tumors occurs in a significant subset of patients, potentially leading to under- or overtreatment if not all lesions are biopsied and profiled [9].

The pathogenesis of TNBC is distinct from hormone receptor-positive tumors, with TNBC often arising via different genetic pathways. Research shows that TNBC is typically associated with TP53 mutations, high genomic instability, and basal-like gene expression profiles. This aggressive phenotype is more commonly observed in younger women and in those with high breast density, both features present in our patient, who was 45 years old at diagnosis and had dense breast tissue reported in imaging studies [6]. However, TNBC is reported to be less prevalent among Caucasian women compared to other ethnic groups, such as African and Hispanic ancestry populations [6,11]. Kumar et al. further emphasized that TNBC is characterized by an atypical risk profile, often unrelated to traditional risk factors such as BMI, smoking, or alcohol consumption [11].

Despite both tumors being classified as T1 and node-negative (stage IA), the presence of an aggressive TNBC lesion warranted an intensified treatment approach. It is emphasized that in cases of SMBC, treatment should not solely be based on tumor size but must also incorporate the most aggressive biological features. The current AJCC staging system, which classifies tumors based on the largest lesion, may not sufficiently reflect the overall tumor burden in MF/MC cases [5,7,10].

One of the most challenging aspects of managing synchronous MF and MC breast cancer is the potential for biological discordance between tumor foci [1,4,8]. While current diagnostic guidelines typically recommend molecular profiling of the largest lesion only [5], this approach may overlook meaningful differences that can influence prognosis and treatment [8,9].

Our patient presented with two tumors of distinct molecular profiles: a luminal A subtype (ER+/PR+/HER2-, Ki-67 10%) and a triple-negative subtype (ER-/PR-/HER2-, Ki-67 30%). This kind of intertumoral heterogeneity has been increasingly recognized in recent literature. In a recent analysis, discordant intrinsic subtypes (as defined by immunohistochemistry and gene expression) were found in 25% of bifocal breast cancers, with clinical implications (e.g., changes in adjuvant therapy) in approximately 8% of cases [4].

Corso et al. reported that histologic and immunophenotypic discrepancies between synchronous lesions occurred in a significant proportion of MC breast cancer cases. These differences may alter therapeutic pathways, especially if only one lesion is sampled [8]. Another study highlighted that current clinical practice might underestimate disease complexity, particularly in cases with discordant hormone receptor and HER2 status across foci. They advocate for more comprehensive sampling in patients with MF or MC presentations [3].

The frequency and significance of these molecular discrepancies were further supported by Mosbah et al., who analyzed bifocal breast tumors and found notable discordance in grade, histology, and receptor expression in up to 36% of paired lesions. Their findings emphasize the risk of basing clinical decisions on a single focus, potentially leading to misclassification of tumor subtype and risk stratification [1].

Lang et al. provided genomic-level insight into this heterogeneity, showing that even histologically similar tumor foci may exhibit diverging gene expression profiles, suggesting polyclonal origins or divergent clonal evolution. Their study supports the hypothesis that MF/MC tumors may not be biologically equivalent and may require individualized therapeutic strategies [12].

In the clinical context, this heterogeneity necessitates receptor status assessment for each discrete tumor. Particularly in patients like ours, where one focus is TNBC, known for its aggressive behavior and lack of targeted therapy [6,11], therapeutic planning must account for the most aggressive component. Relying solely on the index tumor may result in undertreatment or, conversely, overtreatment, especially in node-negative patients where adjuvant therapy decisions heavily depend on molecular subtype [5,8].

In our case, the second tumor, emerging shortly after a VABB, raised concerns about possible iatrogenic seeding. However, VABB is considered safe and effective, with a high technical success rate and minimal complications. In a large cohort, Fang et al. reported hematomas in 10.9% and vasovagal episodes in 2.8% of cases, confirming its low-risk profile even for malignant lesions [13].

However, Loughran and Keeling described cases of tumor cell seeding along biopsy tracts, emphasizing that displaced malignant cells, especially from triple-negative tumors, may occasionally give rise to secondary foci [14]. Though causality cannot be established, this risk may warrant additional caution in patients with discordant synchronous lesions, especially when new foci develop near previous biopsy sites.

Limitations

This case report is based solely on retrospective review of medical records, without access to original imaging or histopathological slides currently available, which limits visual documentation. Genetic testing was restricted to available panels, and thus untested or novel mutations cannot be excluded. As with any single-patient report, findings may not be broadly generalizable but still offer valuable insights into managing biologically discordant MC breast cancer.

## Conclusions

This case highlights the clinical and biological complexity of synchronous MC breast cancer with discordant molecular subtypes within the same breast. The coexistence of a luminal A tumor and an aggressive triple-negative lesion in separate quadrants emphasizes the need for independent pathological assessment of each tumor focus. The emergence of the TNBC component in the biopsy cavity raises the question of tumor seeding following VABB, although this remains speculative. Given the patient's family history and negative BRCA-related testing, the case also underscores the relevance of broader genetic evaluation. Individualized treatment planning should be guided by the most aggressive tumor subtype, and close monitoring of biopsy sites remains essential.
